# Effects of benzydamine and mouthwashes containing benzydamine on *Candida albicans* adhesion, biofilm formation, regrowth, and persistence

**DOI:** 10.1007/s00784-021-04330-8

**Published:** 2022-01-23

**Authors:** Andrea Ardizzoni, Giorgia Boaretto, Eva Pericolini, Diego Pinetti, Alessandra Capezzone de Joannon, Lucia Durando, Lorella Ragni, Elisabetta Blasi

**Affiliations:** 1 Department of Surgical, Medical, Dental and Morphological Sciences with interest in Transplant, Oncological and Regenerative Medicine, University of Modena and Reggio Emilia, Via Campi, 287, 41125 Modena, Italy; 2 Graduate School of Microbiology and Virology, University of Modena and Reggio Emilia, Modena, Italy; 3 Centro Interdipartimentale Grandi Strumenti (C.I.G.S.), University of Modena and Reggio Emilia, Modena, Italy; 4 Global R&D PLCM—Angelini Pharma S.p.A., via Vecchia del Pinocchio 22, 60131 Ancona, Italy

**Keywords:** Benzydamine, Mouthwashes (MoWs), *Candida albicans*, Bioluminescence, Biofilm, Adhesion

## Abstract

**Objectives:**

To assess the effects of benzydamine and mouthwashes (MoWs) containing benzydamine on different stages of *Candida albicans* biofilm: adhesion, formation, persistence, and regrowth (if perturbed).

**Materials and methods:**

*C. albicans* CA1398, carrying the bioluminescence ACT1p-gLUC59 fusion product, was employed. Fungal cells were exposed for 1′, 5′, or 15′ to 4 different benzydamine concentrations (0.075 to 0.6%) to 2 mouthwashes (MoWs) containing benzydamine and to a placebo MoW (without benzydamine). Treated cells were tested for adhesion (90 min) and biofilm formation (24-h assay). Next, 24- and 48-h-old biofilms were exposed to benzydamine and MoWs to assess regrowth and persistence, respectively. The effects of benzydamine, MoWs containing benzydamine, and placebo on different biofilm stages were quantified by bioluminescence assay and by the production of quorum sensing (QS) molecules.

**Results:**

Benzydamine and MoWs containing benzydamine impaired *C. albicans* ability to adhere and form biofilm, counteracted *C. albicans* persistence and regrowth, and impaired a 48-h-old biofilm. Some of these effects paralleled with alterations in QS molecule secretion.

**Conclusions:**

Our results show for the first time that benzydamine and MoWs containing benzydamine impair *C. albicans* capacity to form biofilm and counteract biofilm persistence and regrowth.

**Clinical relevance:**

Benzydamine and MoWs containing benzydamine capacity to affect *C. albicans* biofilm provides an interesting tool to prevent and treat oral candidiasis. Likely, restraining *C. albicans* colonization through daily oral hygiene may counteract colonization and persistence by other critical oral pathogens, such as *Streptococcus mutans*, whose increased virulence has been linked to the presence of *C. albicans* biofilm.

**Supplementary Information:**

The online version contains supplementary material available at 10.1007/s00784-021-04330-8.

## Introduction

*Candida albicans* (*C. albicans*) often colonizes the oral cavity of healthy subjects, where it appears in its yeast form. Because of its dimorphism (an important virulence trait, which allows fungal switch between yeast and filamentous forms), *Candida* behaves as an opportunistic pathogen and upon transition to filamentous forms, it is capable of causing mucosal infections mainly in immunocompromised individuals [1, 2]. Oropharyngeal infections by *C. albicans* do not affect only AIDS patients, but they are often related also to diabetes that, in turn, is associated with xerostomia (a salivary pH disorder which reduces the salivary flow [3]), oral cancer [4], and terminally ill conditions [5]. Formation of hyphae is linked to the expression of characteristic hyphae-associated genes, such as hyphal wall protein 1 (Hwp1), agglutinin-like sequence 3 (Als3), secreted aspartic proteases 4, 5, and 6 (Sap4, 5, and 6), and the hyphae-associated proteins extent of cell elongation protein 1 (Ece1) and hyphal regulated cell wall protein 1 (Hyr1) [6]. Since most of such molecules are adhesins, their interaction with cell surface receptors grants the fungus the capacity to bind efficiently to oral mucosal epithelia as well as teeth surface [7, 8]. Such fungal adhesion, promoted also by hyphae formation, facilitates in turn biofilm establishment. In addition, the presence of hyphae elicits proinflammatory cytokine production and helps the fungus to avoid phagocytosis and/or intracellular killing, ultimately causing tissue damage [9]. Biofilm in particular shelters the fungal cells from the action of immune-mediated defenses [10], antifungal drugs, and disinfectants [11–14].

Interestingly, this enhanced resistance of the sessile form of the fungus is not simply due to a mere physical sheltering, but also to the possibility of the biofilm-embedded fungi to form a community, whose components can communicate with each other through the release and monitoring of low molecular weight hormone-like secreted molecules. Such mechanism is known as *quorum sensing* (QS) and the molecules responsible for the inter-microbial communication are collectively indicated as QS molecules [15]. Four main QS molecules have been described to date in the kingdom of *Fungi*: farnesol, tyrosol, phenylethanol, and tryptophol [16, 17]. Their concentration within the biofilm environment has been reported to be proportional to the size of the fungal population: beyond a critical threshold, a response is triggered, leading to the coordinated expression or repression of QS-related genes [18]. QS molecules can regulate *Candida* morphogenesis (by stimulating yeast-to-hyphae or hyphae-to-yeast switching), can initiate fungal apoptosis, and can affect the fungal virulence [17]. Among QS molecules, farnesol and tyrosol are known to be involved in biofilm formation by modulating several virulence factors of the fungus including its dimorphic transition. In particular, farnesol induces hyphae-to-yeast transition and inhibits biofilm formation, whereas tyrosol has an opposite effect by stimulating hyphae production and therefore exerting a pivotal role in biofilm production [15].

In addition to yeast-to-hyphae switching, *Candida* cell surface hydrophobicity is another important feature that reportedly favors fungal adhesion to inert surfaces. This may represent another explanation for frequent oral infections in those individuals who harbor abiotic materials within the oral cavity for therapeutic purposes (such as acrylic denture base, orthodontic metal braces, and surface of dental restorations) [19–22]. For this same reason, oral candidiasis can easily develop in individuals (especially elderly people) who make use of dental prostheses, as well as in all those individuals/patients who fail to produce sufficient saliva [23].

Good oral hygiene practices, such as daily use of toothbrush, toothpaste, and mouthwash, are crucial in contributing to prevent oral infections by *Candida* [24]. As a matter of fact, we have recently demonstrated, by means of in vitro models, that both *C. albicans* hyphal development and biofilm formation and persistence can be affected by several mouthwashes, especially by those containing chlorhexidine digluconate, cetylpyridinium chloride, and essential oils in their formulations [9, 25].

Benzydamine hydrochloride is a nonsteroidal drug with anti-inflammatory effects mainly due to its capacity to inhibit TNF-α and IL-1β production and release [26, 27]. Also, this molecule has analgesic and anesthetic properties [28–30]. Even though benzydamine cannot be considered an antibiotic, it has been demonstrated to have some antimicrobial effects [31]. Because of its properties, benzydamine has been included in the formulation of several mouthwashes, where its use as an anti-inflammatory drug was mainly intended to delay or prevent radiation-induced oral mucositis in patients undergoing radiation therapy [32–34]. More recently, mouthwashes and oral spray formulations, containing either 0.15% benzydamine hydrochloride or 0.2% chlorhexidine digluconate, were shown to have similar anti-inflammatory effectiveness against gingival inflammation caused by plaque accumulation [35, 36]. Notwithstanding the similarity in the anti-inflammatory activity, benzydamine was generally better tolerated and it lacked the side effects associated with the use of chlorhexidine, such as staining of teeth, loss of taste, and feeling of numbness [37]. Mouthwashes containing a combination of benzydamine hydrochloride and cetylpyridinium chloride were proven effective as well in reducing plaque formation, in comparison with mouthwashes containing cetylpyridinium alone, without any relevant clinical or microbiological adverse effect [38].

Little is known about the antifungal effects of benzydamine hydrochloride on *C. albicans*, especially on the fungus capacity to adhere to substrates and to form biofilm. For this reason, here we performed a series of in vitro studies, in order to assess if benzydamine hydrochloride (either alone or in combination with other molecules within commercial mouthwashes) could impair *C. albicans* adhesion to an abiotic substrate and its capacity to form a biofilm. Moreover, we evaluated the capacity of the molecule (and of the MoWs containing it) to disrupt mature biofilm and to interfere with its persistence and regrowth. We then assessed the effect of MoWs containing benzydamine in the production of *C. albicans* QS molecules, a key factor involved in both biofilm formation and biofilm persistence.

## Materials and methods

### Reagents

Benzydamine (l-benzyl-3-dimethylaminopropoxy-lH-indazole hydrochloride) was supplied by Angelini Pharma S.p.A. (Roma, Italy). The powder was resuspended in complete F12 medium (cF12). The latter consisted of Dulbecco’s modified Eagle’s medium/Ham’s nutrient mixture F12 (Sigma, St. Louis, MO, USA), supplemented with 10% heat-inactivated fetal bovine serum (HiFBS, Defined Hyclone, Logan, UT, USA), 50 mg/ml gentamicin (Bio Whittaker, Verviers, Belgium), 2 mg/ml Ciproxin (ICN Biomedicals, Cleveland, OH, USA), and 2 mM l-glutamine (EuroClone, Milan, Italy). The benzydamine solution was sterilized by filtration with 0.22-μm syringe filters and diluted to the working strength concentrations used for the experiments (see below). Benzydamine was always resuspended and diluted fresh, on the day of the experiment.

Three different mouthwashes were also employed for the study. MoW 1 (“Tantum Verde 0.15% collutorio”, Angelini Pharma S.p.A., Roma, Italy) containing 0.15% benzydamine hydrochloride and 96% ethanol; MoW 2 (“Tantum Verde Bocca 22.5 mg/15 ml + 7.5 mg/15 ml collutorio”, Angelini Pharma S.p.A., Roma, Italy) containing 0.15% benzydamine hydrochloride, 0.05% cetylpyridinium chloride, and 96% ethanol; MoW 3 (supplied by Angelini Pharma S.p.A., Roma, Italy) containing only ethanol and used as a placebo. MoWs 1 and 2 have been commercially available for several years; they are safe and not toxic (further information on this topic can be found in the supplementary materials section).

### Fungal strain

The BLI *Candida albicans* strain employed in this study was the CA1398, carrying the bioluminescence ACT1p-gLUC59 fusion product [39]. To assess the effects of different benzydamine concentrations and contact times as well as the effects of different MoWs employed for different contact times on *C. albicans* viability, fungi were counted to a working strength concentration of 2 × 10^6^/ml and resuspended in cF12 medium (see above).

### MIC determination

The benzydamine MIC for BLI-*Candida* was evaluated according to the broth microdilution method NCCLS M27 [40]. The data obtained were confirmed by reading the optical density (O.D.) at 540-nm wavelength, by means of a plate reader (Tecan Sunrise, Austria).

### Experimental design

#### Protocol for adhesion and biofilm production

In order to evaluate the effects of benzydamine on *Candida* capacity to adhere to plastic and to form a biofilm, fungal cells (2 × 10^6^/ml in cF12 medium) were treated for 1, 5, or 15 min with 4 different concentrations of benzydamine (6 mg/ml (0.6%), 3 mg/ml (0.3%), 1.5 mg/ml (0.15%), and 0.75 mg/ml (0.075%)), with the 3 MoWs, or with cF12 medium alone (used as a control). After treatment, fungal cells were washed, dispensed in a black 96-well microtiter plate (PerkinElmer Life Sciences, Cambridge, UK) (0.1 ml/well, in triplicate), and placed at 37 °C with 5% CO_2_. Incubation was carried out for 90 min (for adhesion assessment) or for 24 h (for biofilm formation assessment).

#### Protocol for biofilm regrowth

To establish the effects of benzydamine on biofilm regrowth, *Candida* cells (2 × 10^6^/ml in cF12 medium) were dispensed in a black 96-well microtiter plate (0.1 ml/well, in triplicate) and placed at 37 °C with 5% CO_2_ for 24 h, to allow biofilm production. Then, *Candida* was exposed to the concentrations of benzydamine indicated above, to the MoWs, or to cF12, for 1, 5, or 15 min. Finally, the wells were gently washed with PBS, 100 μl of fresh cF12 was added to each well, and the plate was placed back at 37 °C with 5% CO_2_ and incubated for additional 24 h, prior to be assessed for microbial regrowth.

#### Protocol for biofilm persistence

**Fig. 1:**
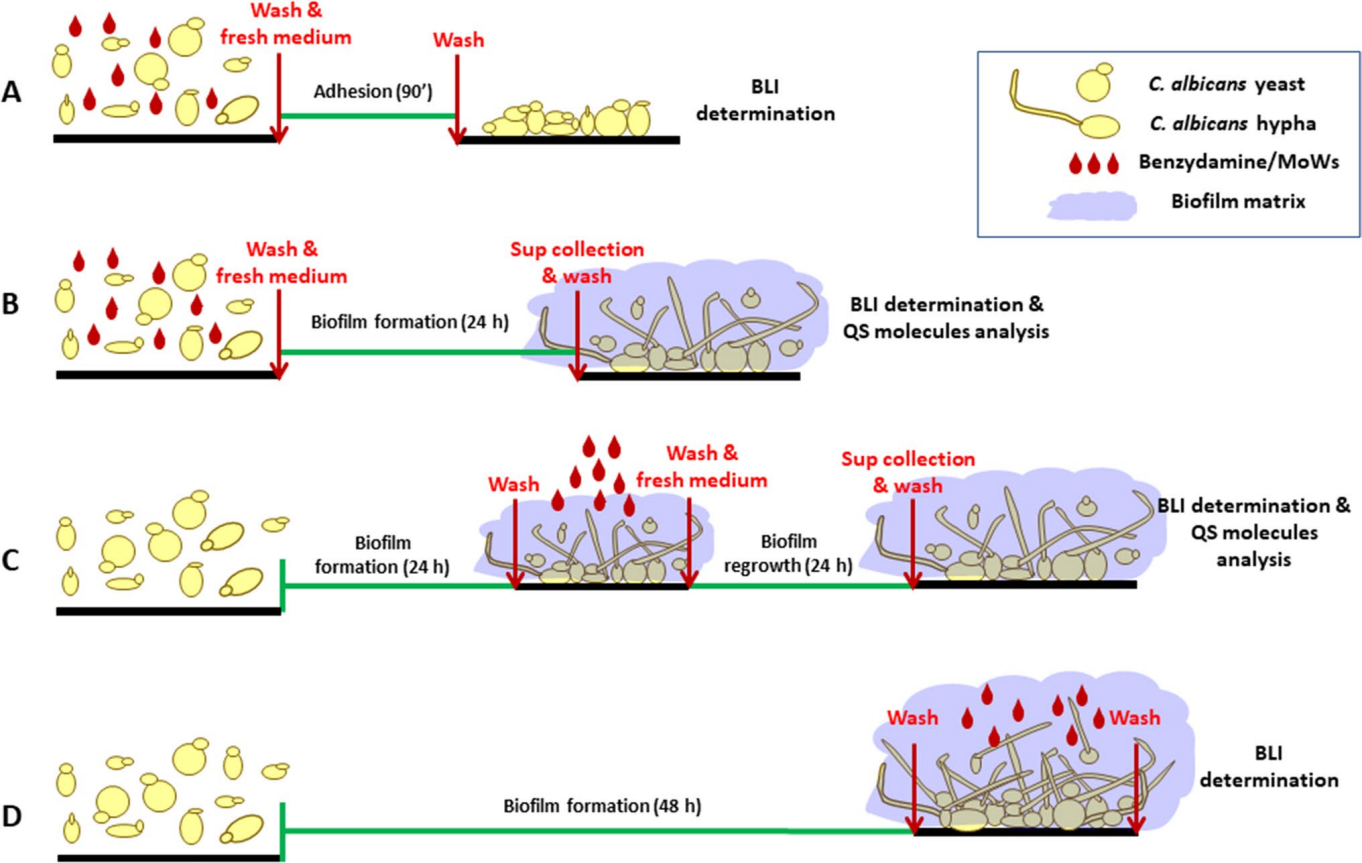
Experimental design performed to assess the effects of benzydamine and MoWs on BLI-*Candida* adhesion (**A**), biofilm formation (**B**), 24-h-old biofilm regrowth (**C**), and 48-h-old biofilm persistence (**D**). BLI-*Candida* cells, incubated for 1, 5, or 15 min with different concentrations of benzydamine or different MoWs, were seeded in 96-well plates. Adhesion was assessed after 90 min of incubation (**A**) and the capacity to form a biofilm was evaluated after 24 h of incubation (**B**). To test the capacity of biofilm regrowth, a 24-h-old biofilm was treated with different concentrations of benzydamine or different MoWs and further incubated up to 48 h (**C**). Finally, the effects of different benzydamine concentrations and different MoWs on biofilm persistence were observed by treating a 48-h-old BLI-*Candida* biofilm (**D**). The results of all these experiments were achieved by bioluminescence assay

To evaluate the effects of benzydamine and MoWs on a mature biofilm, *Candida* cells (2 × 10^6^/ml in cF12 medium) were dispensed in a black 96-well microtiter plate (0.1 ml/well, in triplicate) and placed at 37 °C with 5% CO_2_ for 48 h, to allow production of a mature biofilm. Then, *Candida* was exposed to the concentrations of benzydamine indicated above, to the MoWs, or to cF12 for 1, 5, or 15 min, prior to be assessed for residual biofilm. The timeline of each experimental protocol is detailed in Fig. 1.

### Bioluminescence analysis

After incubation at 37 °C with 5% CO_2_ (carried out for different times, according to the parameter investigated, as detailed in the previous paragraph), the supernatants were collected and stored at −80 °C for the HPLC-ESI/HRMS analysis (see below). Then, the wells were gently washed 3 times with PBS. Then, 2 μM coelenterazine (SynChem, Ohm, Germany) in luciferase assay buffer (LA buffer) was added and the relative luminescence units (RLU) were assessed by immediately reading the plate with a Fluoroskan FL luminometer (Thermo Scientific, Waltham, MA, USA).

### Fungal viability measurement

*Candida* suspensions (0.5 ml/tube) were exposed to benzydamine (0.5 ml/tube; at different concentrations) or to the MoWs (0.5 ml/tube); the tubes were incubated at 37 °C with 5% CO_2_ for 1, 5, or 15 min. As controls, *Candida* suspensions (0.5 ml/tube) were added to 0.5 ml of cF12 and incubated at 37 °C with 5% CO_2_ for the same times. After each time of contact, the tubes were centrifuged at 4500 rpm for 8 min, washed with PBS, centrifuged again, and suspended with cF12. Serial dilutions of *Candida* suspensions were then seeded in SDA plates to evaluate the growth inhibition capacity of different benzydamine concentrations and of the different MoWs formulations per se.

### Assessment of the quorum sensing (QS) molecules by liquid chromatography–electrospray/high-resolution mass spectrometry (HPLC-ESI/HRMS)

After MoWs containing benzydamine treatment, the detection of 2 quorum sensing (QS) molecules, farnesol and tyrosol, was performed by HPLC-ESI/HRMS. Tyrosol (2-(4-hydroxyphenyl)-ethanol) and farnesol (3,7,11-trimethyl-2,6,10-dodecatriene-1-ol) standard solutions were supplied by Sigma-Aldrich (St. Louis, MO, USA). Both reagents were reconstituted to 1 mg/ml in 95% methanol (Carlo Erba Reagents, Milan, Italy). External standard calibration samples were prepared in water:methanol 95:5 (v/v) to cover the 0.5 to 1000 ng/ml concentration range for both analytes.

The levels of tyrosol and farnesol were measured in supernatants collected from untreated controls and samples treated for 15 min with the MoWs, after a further incubation for 24 h (biofilm formation) and 48 h (biofilm regrowth). The supernatants, which had been stored at −80 °C, were thawed and centrifuged at 14,000 rpm for 10 min, in order to get rid of cellular debris. Then, they were transferred to Amicon-Ultra 0.5 tubes (Sigma-Aldrich, St. Louis, MO, USA) and centrifuged again at 14,000 rpm for 15 min. Prior to be analyzed, such samples were diluted 1:1 (v/v) with 5% methanol in water and transferred to the autosampler pending analysis.

Analyses were performed on an Ultimate 3000 HPLC connected to a Q Exactive high-resolution mass spectrometer via a HESI-II electrospray ionization source (Thermo Scientific), controlled by Xcalibur software (Thermo Scientific, v. 29 build 2926). A 10-μl volume of sample solution was injected onto a Hypersil Gold C18 100 × 2.1 mm ID 1.8 μm ps column (Thermo Scientific, Waltham, MA, USA) kept at 30 °C and separation was performed at 0.4 ml/min flow with a gradient elution scheme using methanol (B) and 0.1% formic acid in water (A). The mobile phase composition was kept at 2% B for 0.2 min after injection then linearly raised to 42% B in 15 min and further on to 98% B in 3.3 min. Methanol was kept at 98% up to minute 24.9 then lowered to 2% at minute 25. The total runtime was 35 min. ESI source was operated in positive ionization mode. Capillary temperature was set at 320 °C; the following nitrogen flows (arbitrary units) were used to assist the ionization: Sheath Gas 40, Aux Gas 30 (at 290 °C), Sweep Gas 3. The capillary voltage was set to 3.8 kV and S-Lens RF level was set at 45 (arbitrary units).

Tyrosol and farnesol were monitored in positive ionization mode using targeted SIM (tSIM) experiments with a 3-min window (retention time ± 1.5 min), 0.5 amu isolation window (target *m*/*z* ± 0.25 amu), AGC target value of 2E5, 140000 FWHM resolution (at *m*/*z* 200), and maximum ion injection time of 503 ms. In-source CID at 8 eV was used during tyrosol detection.

The molecular protonated ion ([M+H]^+^) was observed for tyrosol at 121.06479 *m*/*z* while farnesol was detected by means of its most abundant in-source fragment at 205.19508 *m*/*z* due to loss of water from its molecular protonated ion ([M+H-H_2_O]^+^).

### Statistical analyses

All data are from 3 different experiments, with triplicate samples. Quantitative variables were tested for normal distribution by the Shapiro-Wilk test. Statistical differences between groups were analyzed according to the Kruskal-Wallis followed by Dunn’s multiple comparisons test by using GraphPad Prism 8. Values of *p ≤ 0.05, **p ≤ 0.01, ***p ≤ 0.001, and ****p ≤ 0.0001 were considered significant.

## Results

### Effects of benzydamine and MoWs containing benzydamine on *C. albicans* adhesion

**Fig. 2:**
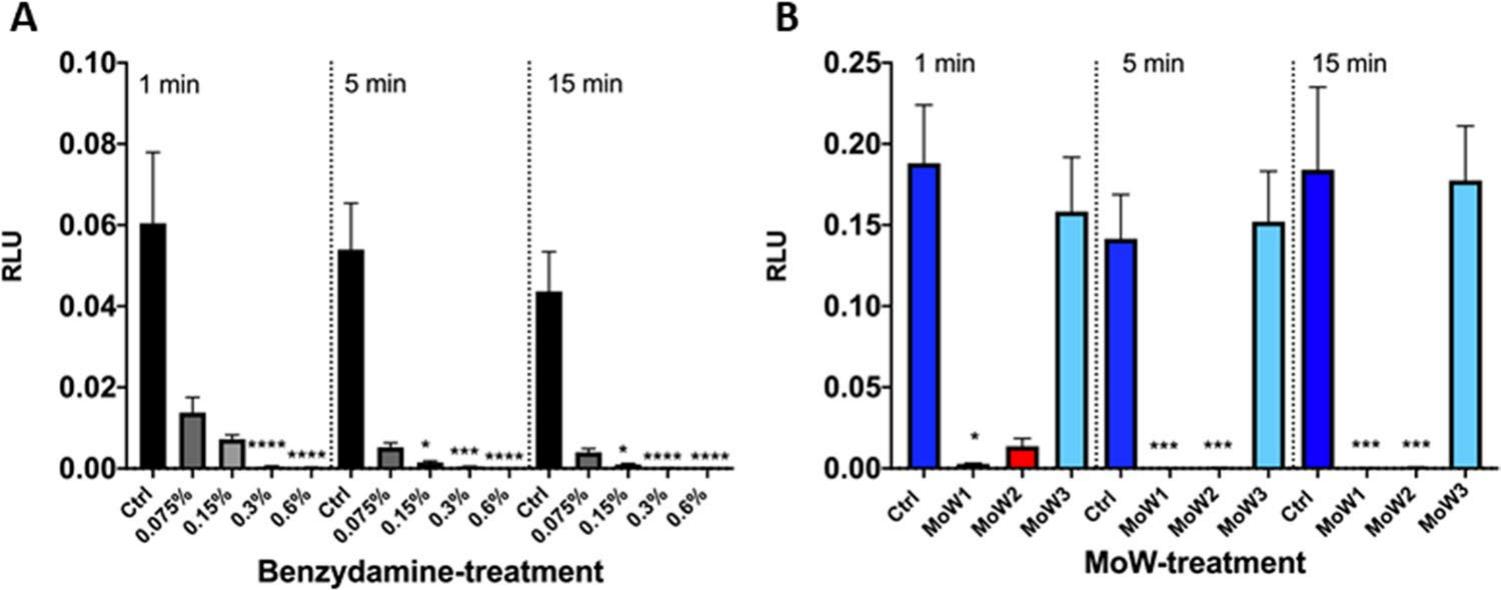
Effects of benzydamine (**A**) and MoWs (**B**) treatments on BLI-*Candida* adhesion. *Candida* cells, pretreated or not with different concentrations of benzydamine or with different MoWs for 1, 5, or 15 min, were assessed for the capacity to adhere to plastic, by bioluminescence assay. The results are shown as mean values ± standard errors of triplicate samples from three different experiments. *p ≤ 0.05, ***p < 0.001, or ****p < 0.0001. RLU, relative luminescence units. MoW 1 (“Tantum Verde 0.15% collutorio”) contains 0.15% benzydamine hydrochloride and 96% ethanol; MoW 2 (“Tantum Verde Bocca 22.5 mg/15 ml + 7.5 mg/15 ml collutorio”) contains 0.15% benzydamine hydrochloride, 0.05% cetylpyridinium chloride, and 96% ethanol; MoW 3 (placebo) contains only ethanol

By using the bioluminescence assay, the fungal capacity to adhere to plastic was assessed, using a previously described protocol [9]. *Candida* cells incubated for 90 min in medium alone (Ctrl) returned the highest bioluminescent signal, indicating the strong capacity of the fungus per se to adhere to the plastic microwells. As shown in Fig. 2A, by pre-treating *Candida* with the highest benzydamine concentrations, i.e., 0.6% and 0.3%, the bioluminescent signals were always reduced in a highly significant fashion, irrespective of the contact times. The pretreatment of *Candida* with 0.15% benzydamine determined a reduction of the bioluminescent signals, which became significant only after 5- and 15-min contact times. Finally, by pre-treating *Candida* with the lowest benzydamine dose, i.e., 0.075%, a consistent (albeit not significant) reduction of bioluminescence signal could be observed.

In a second set of experiments, the effect of commercial MoWs containing 0.15% benzydamine (albeit with different formulations) was assessed in comparison to placebo MoW without benzydamine on *Candida* adhesion to plastic surface. Again, *Candida* cells incubated for 90 min in medium alone (Ctrl) always returned the highest bioluminescent signal. A trend very similar to the control *Candida* samples could be observed when the fungal cells were pretreated with MoW 3, used as a placebo. Differently, the pretreatment of *Candida* with MoWs 1 and 2 caused a highly significant reduction of the bioluminescent signals, irrespective of the contact times (Fig. 2B).

### Effects of benzydamine and MoWs containing benzydamine on the capacity of *C. albicans* to form a biofilm

**Fig. 3:**
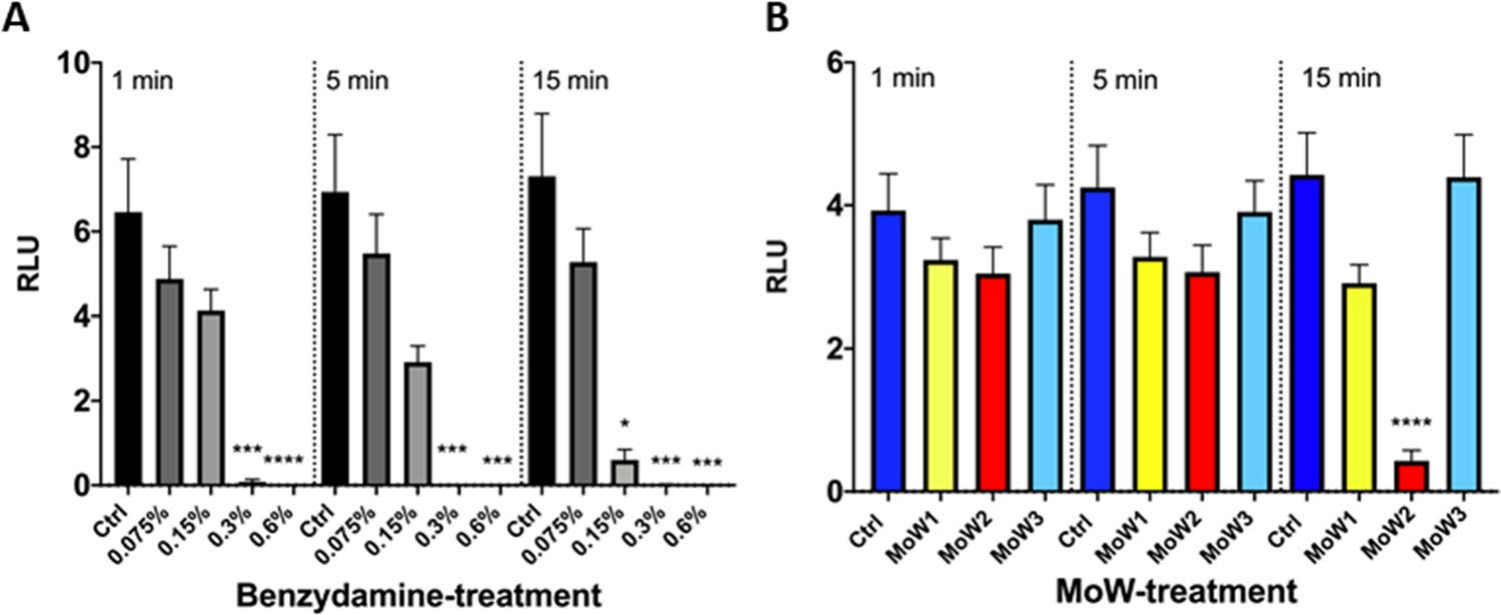
Effects of benzydamine (**A**) and MoWs (**B**) treatments on the capacity of BLI-*Candida* to form a biofilm. *Candida* cells, whether pretreated with different concentrations of benzydamine or with different MoWs for 1, 5, or 15 min, were tested for the ability to form a biofilm by bioluminescence assay. The results are shown as mean values ± standard errors of triplicate samples from three different experiments. *p ≤ 0.05, ***p < 0.001, or ****p < 0.0001. RLU, relative luminescence units

The bioluminescence assay was employed to assess the capacity of benzydamine- and MoWs-pretreated *Candida* cells to produce a biofilm. Fungal cells, incubated for 24 h in medium alone (Ctrl), returned the highest bioluminescent signal, indicating a strong capacity of the fungus per se to efficiently form a biofilm. By pre-treating *Candida* with the highest benzydamine doses, i.e., 0.6% and 0.3%, the reduction in bioluminescent signals was significantly higher compared to the controls, irrespective of the contact times. Moreover, by pre-treating *Candida* with 0.15% benzydamine, the bioluminescent signals were reduced as well, but statistical significance was reached only after 15-min contact time (Fig. 3A).

By pre-treating *Candida* with MoW 1, the bioluminescent signals were partly reduced at 15 min with respect to the control, even though statistical significance could not be reached. Differently, the pretreatment of *Candida* with MoW 2 caused a significant reduction in bioluminescent signals after 15 min of pretreatment, with respect to both control *Candida* and *Candida* treated with placebo MoW 3 (Fig. 3B).

### Effects of benzydamine and MoWs containing benzydamine on the persistence and regrowth of a 24-h-old *C. albicans* biofilm

**Fig. 4:**
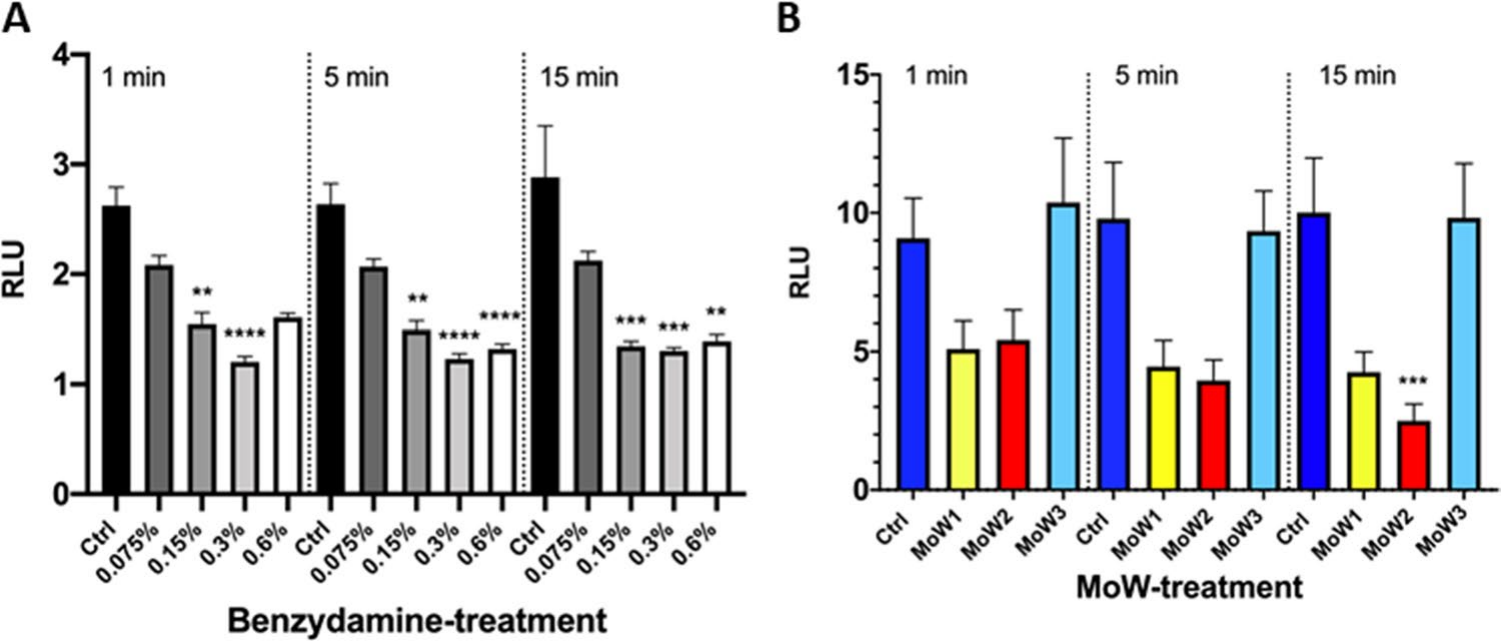
Effects of benzydamine (**A**) and MoWs (**B**) treatments on a 24-h-old BLI-*Candida* biofilm persistence and regrowth. A 24-h-old *Candida* biofilm was exposed to benzydamine (**A**) and MoWs (**B**) treatments, for 1, 5, or 15 min, washed, and further incubated for additional 24 h of incubation at 37 °C. *Candida* regrows in the following 24 h, as assessed by bioluminescence. The results are shown as mean values ± standard errors of triplicate samples from three different experiments. **p < 0.01, ***p < 0.001, or ****p < 0.0001. RLU, relative luminescence units

In order to test how benzydamine and MoWs would affect the capacity of a 24-h-old *C. albicans* biofilm to persist and possibly regrow, we used the experimental approach depicted in Fig. 1C. As shown in Fig. 4A, control *Candida* cells that had been treated with medium alone returned the highest bioluminescent signal at time 48 h, indicating the strong capacity of the fungus to efficiently form a biofilm. When in parallel samples, the 24-h-old *Candida* biofilm had been treated with 0.15%, 0.3%, and 0.6% benzydamine and further incubated for additional 24 h to allow residual biofilm regrowth, the bioluminescent signals were reduced in a highly significant fashion, irrespective of the contact times.

By employing the MoWs containing benzydamine 1 and 2, the decreased bioluminescent signals revealed that the biofilms were indeed consistently affected, no matter whether 1, 5, or 15 min of treatment had been performed; yet statistically significant differences with the controls could be reached only when treating the biofilm with MoW 2 for 15 min. As expected, the treatment of *C. albicans* 24-h-old biofilm with the placebo MoW 3 did not show any difference with the control group (Fig. 4B).

### Effects of benzydamine and MoWs containing benzydamine on a 48-h-old *C. albicans* biofilm

**Fig. 5:**
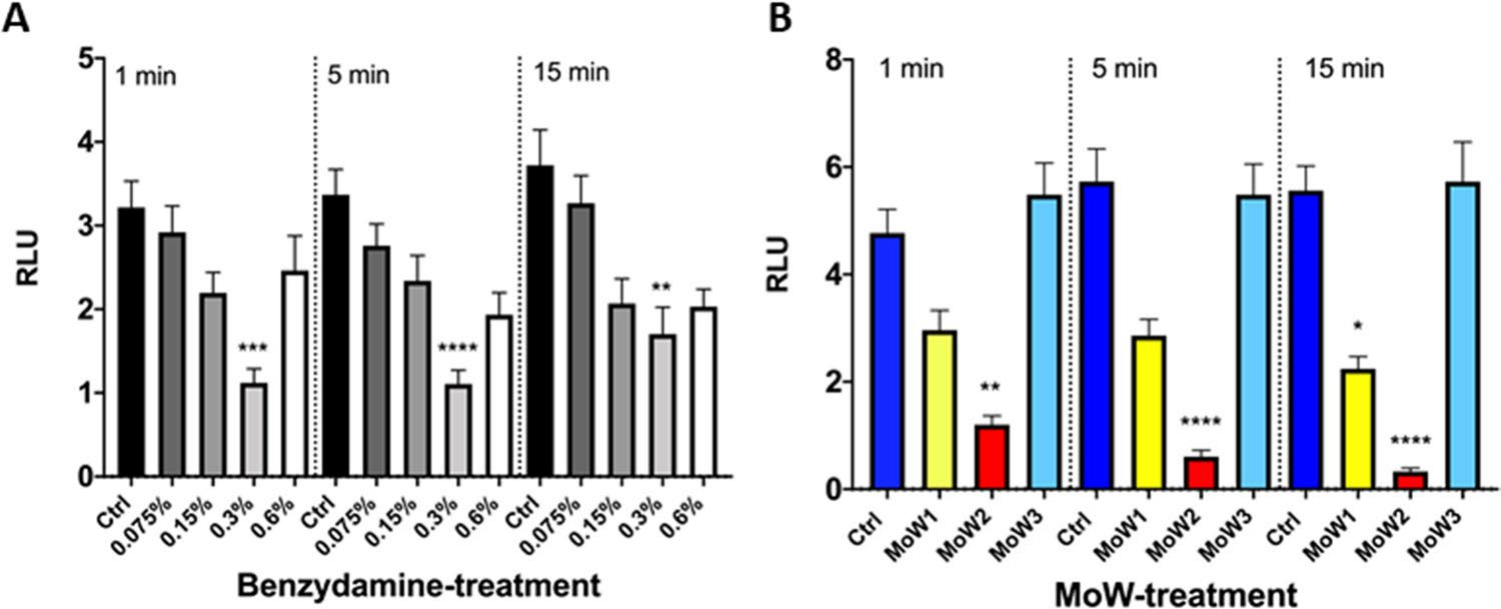
Effects of benzydamine (**A**) or MoWs (**B**) treatments on a 48-h-old BLI-*Candida* biofilm. Benzydamine (**A**) or MoWs (**B**) were used against a 48-h-old *Candida* biofilm for 1, 5, or 15 min; then, the amounts of residual biofilm were assessed by bioluminescence. The results are shown as mean values ± standard errors of triplicate samples from three different experiments. *p ≤ 0.05, **p < 0.01, ***p < 0.001, or ****p < 0.0001. RLU, relative luminescence units

Finally, the effects of benzydamine and MoWs containing benzydamine were assessed on a 48-h-old biofilm. The untreated *Candida* cells, embedded in a 48-h-old biofilm, returned the highest bioluminescent signal, once again confirming the strong capacity of the fungus to form efficiently a mature biofilm. The treatment of such 48-h-old *Candida* biofilm with 0.15% and 0.3% benzydamine returned a dose-dependent decrease in bioluminescent signals, reaching statistical significance at 0.3% benzydamine treatment, whereas the treatment with 0.6% benzydamine produced a partial but non-significant reduction (Fig. 5A).

The treatment of the 48-h-old *Candida* biofilm with the MoWs reduced the bioluminescent signal to a different extent, depending on the MoW used (Fig. 5B). In particular, MoW 1 significantly impaired the preformed biofilm only when used for 15 min; differently, MoW 2 treatment significantly reduced the bioluminescence signal, at all the times; finally, the biofilm treated with MoW 3 consistently returned bioluminescent signals similar to the control samples.

### Effects of benzydamine and MoWs containing benzydamine on fungal viability

**Fig. 6:**
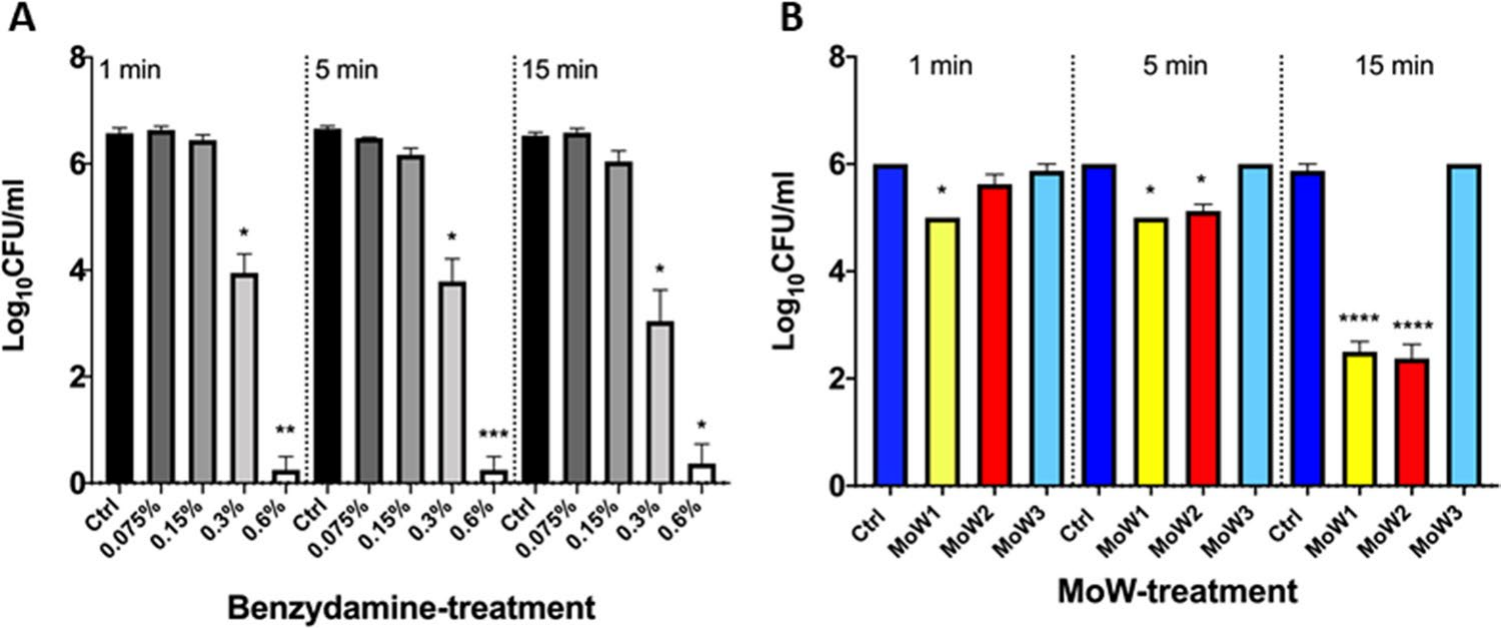
Effects of benzydamine (**A**) or MoWs (**B**) on *C. albicans* viability. Yeast cells were treated for 1, 5, or 15 min with benzydamine (**A**; from 0.075 to 0.6%) and with MoWs (**B**); in all the cases, control cells were incubated in cF12 alone (controls). Then, the cells were washed, diluted with PBS, and seeded in SAB agar plates where they were let to grow at 37 °C. After 48 h, the CFU were counted, and the results were expressed as mean values of Log (CFU/ml) ± standard errors of duplicate samples from three different experiments. *p ≤ 0.05, **p < 0.01, ***p < 0.001, or ****p < 0.0001 indicate significant differences between benzydamine/MoWs-treated vs. cF12-treated *C. albicans*

In order to verify if the contact with benzydamine or MoWs containing benzydamine could decrease fungal cell viability, the CFU counts were evaluated after contact between *Candida* and benzydamine at various concentrations or MoWs. The results showed that, at the highest concentration tested (0.6%), benzydamine significantly inhibited fungal viability, irrespective of the contact times, whereas the treatment with 0.3% benzydamine reduced fungal viability of 2 log at all the contact times. No significant effects on fungal viability were observed after treatment with the lowest benzydamine concentrations, i.e., 0.15% and 0.075%, irrespective of the contact times (Fig. 6A).

The CFU counts after contact with the MoWs were also evaluated. The results showed that MoW 1 significantly inhibited fungal viability of about 1 Log, after 1 and 5 min of contact time, whereas a more pronounced effect on fungal viability could be observed after 15-min contact time (about 3 Log). The treatment with MoW 2 significantly reduced fungal viability of 1 and 3 Log, only after 5- and 15-min contact times, respectively. No significant effect on fungal viability was observed after treatment with the placebo MoW 3, being the CFU counts similar to those observed in the control group (Fig. 6B).

Then, the benzydamine MIC on BLI-*C. albicans* planktonic form was measured, according to the broth microdilution method NCCLS M27 [40]. We found that the MIC value (0.0075 mg/ml) corresponded to the lowest benzydamine dose employed in the above-described experiments (0.075%). Such MIC value was confirmed also by the O.D. reading.

### Effects of MoWs containing benzydamine on quorum sensing (QS) molecules

In order to unravel the mechanisms behind the inhibition of biofilm formation and biofilm regrowth by MoWs, we analyzed the levels of two *Candida* quorum sensing (QS) molecules, tyrosol and farnesol, by HPLC-ESI/HRMS.

**Fig. 7:**
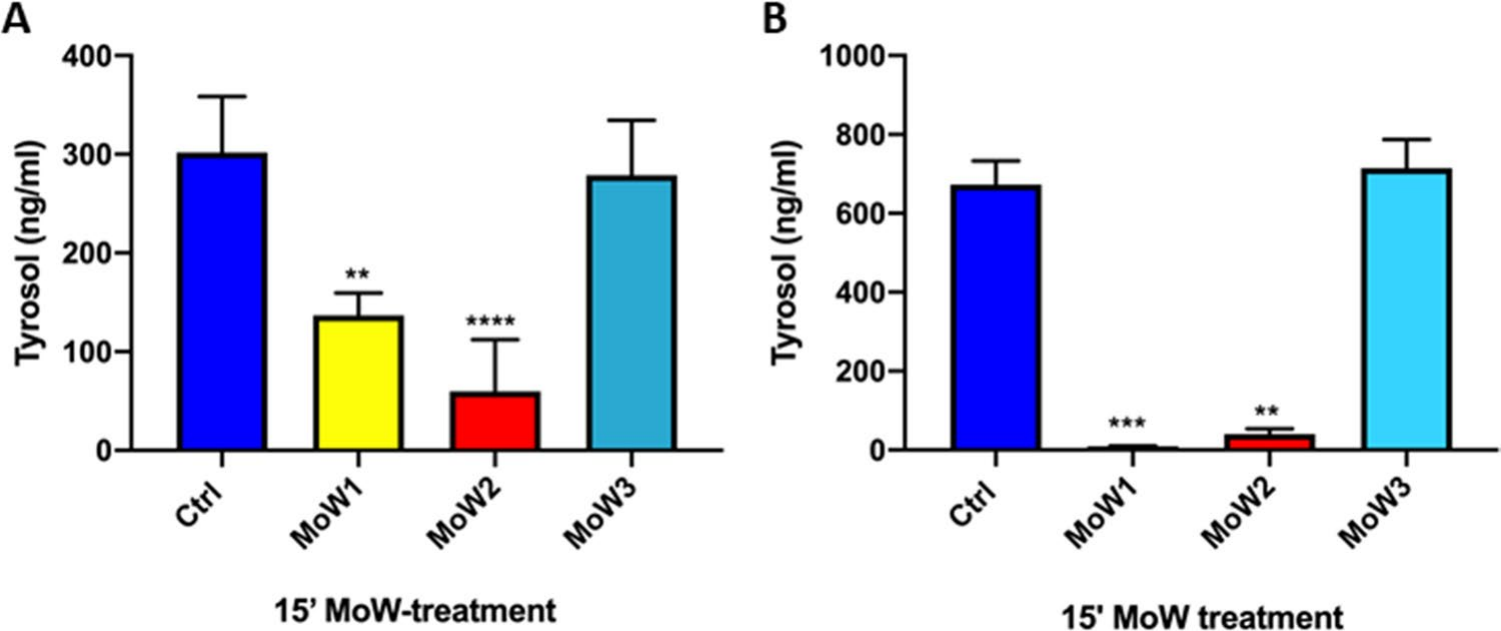
Effects of MoWs on the production of tyrosol by *Candida* biofilm: early (**A**) and late treatment (**B**). By HPLC-ESI/HRMS analysis, the levels of tyrosol were assessed on supernatants from a 24-h-old biofilm formed by *Candida* pretreated for 15 min with the different MoWs (**A**). The tyrosol production after biofilm regrowth was assessed on a 48-h-old *Candida* biofilm that had been treated at 24 h with the different MoWs for 15 min (**B**). The results are shown as mean values ± standard errors of triplicate samples from three different experiments. **p < 0.01, ***p < 0.001, or ****p < 0.0001

Our results show that 15-min treatment with MoWs 1 and 2 significantly impaired the production of tyrosol by *Candida* both during biofilm formation (Fig. 7A) and in the regrowth of a biofilm treated at time 24 h and further incubated in fresh medium up to 48 h (Fig. 7B). Differently, the treatment of *Candida* cells or *Candida* biofilm with the placebo MoW 3 did not impair tyrosol production, whose levels were comparable to those of the control samples.

In contrast, the analysis of farnesol levels consistently returned negative results (data not shown). This may be due to technical bias: either farnesol elution occurred very late, almost at the end of the process, and therefore it was not possible to reveal its signal, or the amounts of *Candida*-released farnesol were too low to be detected by the system.

## Discussion

*C. albicans* is a fungal species, frequently colonizing the skin, the oral cavity, and the mucosal surfaces of several body sites in humans. Certain conditions, such as immunodeficiency [1, 2], diabetes [3], nutritional deficiencies [41], presence of dental prostheses [42], chemotherapeutic treatments [4, 5], and systemic steroid use [43], all contribute to turning this commensal fungus to an opportunistic pathogen [2, 44]. In particular, the capacity of *C. albicans* to adhere to biotic and abiotic surfaces represents the first and crucial step leading to biofilm formation [22, 45]. By organizing itself as a sessile structure, *C. albicans* enhances its pathogenic potential, by increasing its resistance to immune-mediated defenses, to disinfectants, and antifungal drugs as well [10–14]. Interestingly, it has been recently demonstrated that the extracellular polysaccharides of *C. albicans* biofilm facilitate *Streptococcus mutans* colonization, persistence, and biofilm formation [46]. This ultimately suggests that the presence of the fungus in the oral cavity may contribute to enhance the risk of dental caries. Hence, the idea that by restraining *Candida* colonization through daily oral hygiene procedures (i.e., use of toothpaste, MoW, and dental floss) may help counteract also the colonization and persistence of specific oral pathogens, such as the cariogenic microorganism *S. mutans*.

Benzydamine hydrochloride, a nonsteroidal drug known since the early 1960s, is known to have anti-inflammatory, analgesic, anesthetic, and some antimicrobial activities. In addition, safety analysis of benzydamine, administered in different pharmaceutical forms (MoWs, oral spray, and lozenges), did not show clinically important side effects, thus confirming its known safety. For all these reasons, benzydamine has been included in several formulations for topical use, including mouthwashes (see supplementary materials for further details). Indeed, several studies have shown that it can help delay and prevent the development of mucositis. Importantly, its anesthetic properties allow it to reduce also the intensity of pain [32–34, 47]. Several studies have demonstrated that benzydamine-, chlorhexidine digluconate-, and cetylpyridinium chloride-containing MoWs have comparable anti-inflammatory effects, likely because all these molecules have the capacity to reduce plaque-induced gingival inflammation as well as plaque accumulation [36, 38]. Notwithstanding the well-known anti-inflammatory properties of benzydamine, little information is available on its antimicrobial activity. Pina-Vaz and coworkers [31] reported that benzydamine, similarly to other local anesthetics, has some fungistatic or fungicidal effects (depending on the doses) on several species of *Candida*, including *C. albicans*. However, such studies had been carried out on planktonic fungal cells, which are known to be more susceptible than biofilm to drugs. To date, no studies are available on benzydamine effects against *C. albicans* biofilm. In the light of this scenario, here we evaluated the antifungal activity of benzydamine and MoWs containing benzydamine against *C. albicans*, by analyzing all the stages leading to biofilm formation and persistence. Specifically, we analyzed the capacity of benzydamine and MoWs containing benzydamine to interfere with *C. albicans* adhesion, biofilm formation, and biofilm regrowth and persistence. Moreover, the effect on the preformed biofilm structure was also assessed. Since commercial MoWs contain either 0.15% or 0.3% benzydamine, such concentrations were chosen for the present study, in addition to lower (0.075%) or higher (0.6%) doses of the molecule. In addition, it is worth noting that the benzydamine MIC (0.075%) does not have any effect on *Candida* yeast-to-hyphae transition and biofilm formation (data not shown). Accordingly, such concentration is the lowest employed in the present investigations.

The data presented here show that the treatment with benzydamine significantly impairs *C. albicans* capacity to adhere to plastic at all the contact times, when using 0.3% and 0.6% benzydamine. Differently, 5- and 15-min contact times have been necessary to impair *C. albicans* adhesion significantly when using 0.15% benzydamine. This result has been always consistent and, indeed, such impairment in adhesion capacity was observed when using benzydamine hydrochloride, both alone and included the formulations of MoWs 1 and 2. These in vitro data open to the possibility that, if also occurring in vivo, this benzydamine-mediated phenomenon may help prevent oral fungal colonization and persistence, especially in subjects, such as elderly denture wearers, quite frequently developing oral candidiasis [19, 20, 48].

The treatment of *C. albicans* with benzydamine inhibits significantly also biofilm formation at all the contact times, when using 0.3% and 0.6% benzydamine; differently, 15-min contact time has been necessary to significantly inhibit biofilm formation when using 0.15% benzydamine. Such result, showing the impairment of biofilm formation when using the highest doses of benzydamine, is in line with the data obtained in the adhesion assay. This is not surprising, since the capacity of *C. albicans* to adhere to surfaces (either biotic or abiotic) is the first stage of fungal colonization [22]; indeed, it easily allows local growth and leads to biofilm formation [45]. By assessing in parallel the effects of MoWs containing benzydamine on biofilm formation, both MoWs 1 and 2 have shown to be effective in reducing biofilm but only after 15-min contact time, yet the statistical significance is only achieved with MoW 2. This latter result is not surprising since MoW 1 contains only benzydamine, while MoW 2 formulation includes cetylpyridinium chloride and benzydamine that, by acting in combination, likely achieve a better performance.

The 15-min contact time was chosen to verify the impact of benzydamine and MoWs on the production of QS molecules by *Candida*. The significant reduction in tyrosol, observed after treatment with both MoWs 1 and 2, is in line with the results obtained by bioluminescence analysis and provides initial evidence that MoWs containing benzydamine are able to interfere in *C. albicans* QS system, at least in tyrosol production, in turn affecting the capacity of the fungus to form a biofilm.

To further investigate the impact of benzydamine on *C. albicans* biofilm, we used both the molecule alone and MoWs containing benzydamine against a preformed sessile fungal community (24 h old) and a mature (48 h old) biofilm. When analyzing the 24-h-old biofilm, a significant reduction has been found, by using benzydamine at any dose, or the MoWs 1 and 2, the latter providing the best effects. In parallel, the MS analysis of the corresponding supernatants reveals that the tyrosol production drastically drops upon treatment. This result implies that the MoWs damage *C. albicans* QS system in a very specific manner. Indeed, the lack of effects observed when treating with the placebo MoW 3 confirms that *C. albicans* QS system is affected by benzydamine and/or cetylpyridinium. To a similar extent, the 48-h-old *C. albicans* biofilm, upon treatment with 0.15%, 0.3%, and 0.6% benzydamine, displays a significantly reduced viability. In addition, MoWs 1 and 2 show a significant effect in damaging a 48-h-old biofilm at all contact times, with the MoW 2 having the strongest effect. Again, the internal control, placebo MoW 3, has no effects on the mature biofilm.

As assessed by CFU analysis, *C. albicans* cells are not killed by benzydamine, even though a significant decrement in viable cells can be observed upon treatment with 0.3% and, even more, with 0.6% benzydamine. To our opinion, these results argue that the decrement observed in adhesion and capacity to form biofilm stems from the impairment of such fungal virulence traits, rather than from *C. albicans* viability. From here, it may be speculated that benzydamine, as well as MoWs containing benzydamine 1 and 2, do not kill *C. albicans*, but they rather mitigate its virulence turning it back to a harmless commensal.

Overall, by means of a highly sensitive BLI-assay and HPLC-ESI/HRMS analysis of QS molecules, we have shown that *C. albicans* biofilm, in its different stages, is deeply impaired by benzydamine and MoWs containing benzydamine. Such effects happen to be achieved by affecting biofilm-embedded fungal cell virulence and metabolism, as demonstrated by the impaired production of the QS molecule tyrosol. These results are in line with the bioluminescence results, indicating that the inhibition of biofilm formation and biofilm regrowth could be explained by the reduced production of QS molecules, such as tyrosol, suggesting a selective role of MoWs in reducing *Candida* activity and metabolism. These findings may have relevant clinical implications, since the biofilm structure works as a shelter, providing the fungus with a protected environment, where resistance to immune-mediated defenses is enhanced [10] and susceptibility to antifungal drugs (such as fluconazole and amphotericin B) and disinfectants (like chlorhexidine) is greatly reduced [11–14].

In the light of this scenario, the present study documents the ability of benzydamine and MoWs containing benzydamine to counteract *C. albicans* biofilm formation as well as its regrowth and persistence. Such ability may provide an additional advantage for the overall oral health because it might interfere with the persistence of cariogenic pathogens within the oral cavity. Indeed, it has been reported that *C. albicans* and the cariogenic species *S. mutans* often coexist within the same dental biofilm, suggesting the establishment of a strict fungal-bacterial interaction, which in turn would facilitate the occurrence of dental caries [49–56]. Clinical studies further support the existence of a positive correlation between the prevalence of *C. albicans* in the oral cavity and the presence of *S. mutans* as well as the occurrence of dental caries in children [51, 57, 58].

## Conclusion

Benzydamine (both alone and included in MoWs) not only impairs fungal adhesion and biofilm formation, but it also affects biofilm persistence/regrowth and mature biofilm as well. These preliminary data have been obtained only from in vitro studies. If such results will be confirmed by future in vivo studies, MoWs containing benzydamine will provide an aid to prevent oral candidiasis, as well as a possible therapeutic tool to locally treat biofilm-related *Candida* infections. This possibility would become particularly important in several categories of patients, such as elderly denture wearers, immunocompromised individuals, and in general all those categories highly susceptible to develop oral *Candida* infections. Finally, given the positive interplay occurring between *Candida* and *S. mutans* in mixed biofilms, we may envisage that, by its ability to counteract *C. albicans* biofilm, benzydamine may also promote oral health indirectly by interfering with the colonization and persistence of cariogenic pathogens.

## Supplementary Information

Below is the link to the electronic supplementary material.

Supplementary file1 (DOCX 15 KB)
