# Supplementary material for: Effects of benzydamine and mouthwashes containing benzydamine on Candida albicans adhesion, biofilm formation, regrowth, and persistence
Source: Clin Oral Investig. 2022 Jan 23;26(4):3613–25. doi: 10.1007/s00784-021-04330-8 (PMC8979862; doi:10.1007/s00784-021-04330-8)
Supplement: Supplementary file 1 — Supplementary file1 (DOCX 15 KB) [file 784_2021_4330_MOESM1_ESM.docx]

**Supplementary material**

**Toxicity and safety of benzydamine**

The tolerability profile of benzydamine is based on the analysis of clinical safety data obtained by clinical trials and by spontaneous reported adverse events. Safety analysis of this drug administered in different pharmaceutical forms (mouthwash, oral spray, and lozenges) did not show clinically important side effects, confirming thus its known safety.

Several clinical studies were performed to investigate benzydamine efficacy in the treatment of sore throat and painful inflammatory conditions of oropharynx tract, odonto-stomatologic disorders, and other oropharyngeal inflammatory conditions (post-operative sore throat caused by endotracheal and nasal-gastric intubation, post-tonsillectomy pharyngitis and oral radiation mucositis). The safety profile of benzydamine is based on the analysis of the above clinical studies performed since the 1980s; some of these studies were also published (see the list of additional references below).

In these trials, patients received benzydamine mouthwash, oral spray or lozenges for a period that ranged from 1 day to 5 weeks and the benzydamine’s adverse events were of mild or moderate severity confirming thus its known good safety and tolerability. Moreover, an open study involving 7,618 patients with oropharyngeal diseases was performed with the aim of monitoring the frequency of side effects after administration of benzydamine mouthwash. According to the results obtained from such study, benzydamine resulted very well tolerated. No serious adverse events were reported, while only 340 out of the 7,618 patients (= 4.5%) reported slight side effects. Numbness, furry tongue, paraesthesia and burning were the most frequent and these adverse events were ascribed to the local anaesthetic effect of benzydamine responsible for the prompt relief of oral painful conditions. (Benzydamine Investigator Brochure’s Angelini 2019).

Benzydamine is responsible of toxic effects (excitement, convulsions, sweating, ataxia, tremors, and vomiting) only in case of accidental ingestion of large quantities (> 300 mg). Considering the low concentration of this drug in oral preparations, very few cases of accidental or voluntary poisoning due to benzydamine have been reported.

All safety available data arising from any source regarding benzydamine oral-mucosal, vaginal and cutaneous marketed formulations collected in the Periodic Safety Update Report (PSUR) between November 1^st^, 2015, and October 31^st^, 2018 (**Dionisio 2019 -** next issue in 2022) confirmed the benzydamine safety profile and its positive risk/benefit ratio in the approved indications.

**Additional references**

Chang JE et al, 2015, Can J Anaesth, doi: 10.1007/s12630-015-0432-x.

Engels I, 1980, Med Velt, PMID: 7207121

Epstein and Stevenson-Moore, 1986, Oral Surg Oral Med Oral Pathol, doi: 10.1016/0030-4220(86)90035-6.

Epstein et al, 1989, Int J Radiation Oncology Biol Phys, doi: 10.1016/0360-3016(89)90964-4.

Epstein et al, 2001, Cancer, doi: 10.1002/1097-0142(20010815)92:4<875::aid-cncr1396>3.0.co;2-1

Hung et al, 2010, Anesth Analg, doi: 10.1213/ANE.0b013e3181e6d82a.

Hung et al, 2010, Anesth Analg, doi: 10.1213/ANE.0b013e3181d4854e.

Raj and Wickham, 1986, Laryngol Otol, DOI: 10.1017/s0022215100099187**.**
